# Skin injuries in newborns hospitalized in neonatal intensive care: a cross-sectional study*

**DOI:** 10.1590/1980-220X-REEUSP-2024-0058en

**Published:** 2024-08-02

**Authors:** Carolina Tenfen, Grasiely Masotti Scalabrin Barreto, Neide Martins Moreira, Helder Ferreira, Adriana Zilly, Rosane Meire Munhak da Silva

**Affiliations:** 1 Hospital Universitário do Oeste do Paraná, Programa de Residência Multiprofissional em Neonatologia. Cascavel, PR, Brazil.; 2 Universidade Estadual do Oeste do Paraná, Centro de Educação, Letras e Saúde, Foz do Iguaçu, PR, Brazil.; 3 Universidade Estadual do Oeste do Paraná, Centro de Educação, Letras e Saúde, Programa de Residência Multiprofissional em Neonatologia, Cascavel, PR, Brazil.

**Keywords:** Wounds and Injuries, Skin, Infant, Newborn, Intensive Care Units, Neonatal, Neonatal Nursing, Heridas y Lesiones, Piel, Recién Nacido, Unidades de Cuidado Intensivo Neonatal, Enfermería Neonatal

## Abstract

**Objective:**

To analyze the skin injuries of hospitalized newborns and identify factors related to the number of lesions.

**Method:**

This was a cross-sectional epidemiological study carried out over a period of one year in a Neonatal Intensive Care Unit in the west of Paraná. The study included 74 newborns with a score ≥5 on the Newborn Skin Condition Scale. Data analysis by chi-square and Pearson's test (p < 0.05).

**Results:**

The frequency was 25.4%, 59.4% had more than one lesion, mainly dermatitis and pressure injury. Birth characteristics were not related to the number of lesions. Not using antibiotics and parenteral nutrition, hemoglobin >11g/dl, phototherapy, pain score <4 and hospitalization >30 days were related to the number of lesions. The presence of two injuries led to longer healing time and three to longer hospitalization. A higher score on the Skin Condition Scale was related to healing time and late start of the diet.

**Conclusion:**

Skin injuries were found to be infrequent among newborns, but there is still a need to improve practices to prevent and maintain skin integrity.

## RESUMEN

**Objetivo:**

Analizar las lesiones cutáneas en recién nacidos hospitalizados e identificar los factores relacionados con el número de lesiones.

**Material y método:**

Estudio epidemiológico transversal realizado durante un año en una unidad de terapia intensiva neonatal del oeste de Paraná. Fueron incluidos 74 recién nacidos con puntuación ≥5 en la Escala de Estado de la Piel del Recién Nacido. Los datos se analizaron mediante chi-cuadrado y test de Pearson (p < 0,05).

**Resultados:**

La frecuencia fue del 25,4%, el 59,4% presentaba más de una lesión, principalmente dermatitis y lesiones por presión. Las características del nacimiento no se relacionaron con el número de lesiones. No utilizar antibióticos ni nutrición parenteral, hemoglobina >11g/dl, fototerapia, puntuación del dolor <4 y hospitalización >30 días se relacionaron con el número de lesiones. La presencia de dos lesiones conllevaba un mayor tiempo de curación y tres una hospitalización más prolongada. Una puntuación más alta en la escala de estado de la piel se relacionó con el tiempo de curación y el inicio tardío de la dieta.

**Conclusión:**

Se observó que las lesiones cutáneas son poco frecuentes entre los recién nacidos, pero sigue siendo necesario mejorar las prácticas de prevención y mantenimiento de la integridad de la piel.

## INTRODUCTION

The skin is the largest human organ, being responsible for important functions in the body. Newborn babies, especially premature babies, have peculiar characteristics, with structural deficiencies being inversely proportional to gestational age, which leads to problems related to dehydration, thermal instability, electrolyte imbalances, delayed maturation, greater susceptibility to the development of trauma and injuries^(1)^. Preterm babies’ skin is 30% thinner and is not fully developed until 34 weeks of gestational age. These characteristics make it more fragile, causing irritations, allergies and infections more easily^(2)^.

Many premature and low birth weight newborns require hospital care to meet their oxygenation, circulation and metabolic needs. This requires invasive and non-invasive interventions, which on the one hand guarantee the survival of these babies, but on the other cause numerous adverse events. Among these events caused by care at the beginning of life are skin lesions^(3)^.

When newborns are hospitalized, they are often subjected to procedures that require asepsis and antisepsis, baths (hygiene), laboratory tests and dressings, as well as using various devices, such as orotracheal tubes, nasal cannulas (prongs), probes, catheters, sensors, diapers and attachments, which, in direct contact with the skin, make it susceptible to the development of injuries^(4)^.

The prevalence of skin injuries in newborns is high, around 80% develop some type of skin injury in the first month of life, 25% of all premature and low birth weight babies are diagnosed with early-onset sepsis, with non-integral skin being the main gateway^(5)^. A study carried out in Italy revealed that 70% of babies hospitalized in intensive care, both premature and full-term, had at least one alteration to their skin^(6)^.

One of the challenges for the multi-professional team is to ensure patient safety, especially in Intensive Care Units (ICUs), since they contain more severely ill patients and therefore more adverse events due to clinical instability, a high number of interventions and invasive and non-invasive devices^(7)^.

The prevention and care of newborns’ skin is largely related to nursing care, as this professional has direct contact with babies in order to carry out their daily tasks, which are necessary for their survival and health recovery. To this end, their care practice needs to be improved every day, using tools such as the Systematization of Nursing Care to guide the care to be provided by the nursing team, providing humanized and quality care^(7,8)^.

The daily inspection of the skin, which is part of the nurse’s care practices, can strengthen the preservation and protection of the newborn’s skin, which requires the use of technological devices. To do this, nurses must seek out knowledge and evidence-based practices that help systematize care, contributing to safety and quality, and especially to minimizing discomfort, pain and length of hospital stay^(9)^. In order to assess the integrity of newborns’ skin, the literature indicates the use of scales, such as the Newborn Skin Condition Scale (NSCS), which allows for the early detection of lesions, standardizes the assessment by health professionals and individualizes care^(10)^.

Considering the importance of maintaining the integrity of newborns’ skin for their full recovery, this study asked the following questions: What is the incidence and what are the main skin injuries in newborns hospitalized in neonatal intensive care? What factors potentiate their recurrence? The aim of this study was to analyze skin injuries in hospitalized newborns and identify factors related to the number of lesions.

## METHOD

### Study Design

This is a cross-sectional epidemiological study applying the Strengthening the Reporting of Observational Studies (STROBE) guidelines for observational studies.

### Study Site

The study was carried out in the neonatal ICU of a university hospital in the western region of the state of Paraná. This Neonatal ICU has ten beds structured for the care of critically ill newborns, and of these beds, one is organized for cases requiring isolation, regardless of their origin. Most of the newborns admitted to the unit come from the institution’s own Obstetric Center and Neonatal Intermediate Care Unit, but it also admits newborns from other municipalities belonging to the tenth Regional Health Department in the state of Paraná.

### Selection Criteria

The study included newborns hospitalized in the Neonatal ICU, regardless of gestational age and/or admission diagnosis, who at some point had a score of 5 or more on the NSCS. It should be noted that this scale is carried out daily by the unit’s nurse. Newborns who needed to be transferred to another neonatal ICU without the skin injury being treated and those who remained hospitalized after the end of the data collection period were excluded.

#### The Nscs

The NSCS was cross-culturally adapted and validated for use in Brazil in 2012, based on the American Neonatal Skin Condition Score (NSCS), the only scale already validated in the United States of America for use in the neonatal population. This is divided into three topics to be assessed: dryness, erythema and rupture/injury, within these, subtopics, in dryness: a value of 1 is assigned for normal skin, no signs of dry skin, 2 for dry skin, visible peeling and 3 for very dry skin, cracks/fissures; in erythema: value 1 is assigned for no evidence of erythema, 2 for visible erythema, <50% of body surface and 3 for visible erythema, ≥50% of body surface; on rupture/injury: value 1 is assigned for none visible, 2 for small in localized areas and 3 for extensive. The minimum and ideal score is 3 and the worst result is 9^(10)^.

### Data Collection

Data collection took place over a period of one year, starting in October 2022 and ending in October 2023, through a search of electronic medical records by a nurse resident in the neonatology unit. The collection began after the implementation of the NSCS in the Tasy® system and training of the unit›s nursing team by the collector, and then, from the moment the first newborn received a score of 5 or more on the NSCS, the collection began.

The study used a structured instrument organized by the researcher in charge, who has experience in the field and in neonatology research. The following variables were included: i) Birth data: date of birth, gender, birth weight, gestational age, Apgar scores in the first and fifth minute; ii) Hospitalization data: date of hospitalization, complications, days of life when diet was started, use of parenteral nutrition and outcome: discharge or death; iii) Initial injury data: days of life at the onset of the injury, weight, type of injury, NSCS score, infection (present or absent), antibiotics used, laboratory tests (hemoglobin and hematocrit), origin of the injury, stay in phototherapy, Neonatal Infant Pain Scale (NIPS) score^(11)^; iiii) Evolution of the injury: whether there was a worsening of the injury, recurrence of the injury (yes or no).

### Data Analysis and Processing

The data was organized in Excel® spreadsheets by independent double entry. For data analysis, the variables studied were organized in double entry tables and absolute and relative frequencies were calculated. The analysis sought to identify and relate aspects of birth, evolution and the appearance of new skin injuries in the newborn. The chi-square test was used with a significance level of 5% to verify this association. Pearson’s correlation analysis was also carried out between the NSCS score and the birth and skin injury variables, using the XLStat2014® program.

### Ethical Aspects

The research was cleared by the University’s Research Ethics Committee, opinion no. 5.656.829 in 2022 and complied with Resolution 466/2012 of the National Health Council, which regulates research with human beings in Brazil. The consent of the parents responsible for the newborn was requested and, after explaining the objectives of the research and their agreement, the Free and Informed Consent Form was signed in two copies. The confidentiality of the information was ensured by coding the newborns.

## RESULTS

During the study period, 291 newborns were hospitalized in the neonatal ICU and 25.4% (n = 74) had some kind of skin lesion. Of these, 59.4% (n = 44) had more than one type of lesion, the main ones identified being dermatitis and pressure injury.

Most of the newborns with skin injuries were male, with a gestational age of between 32 and 34 weeks and six days and an Apgar score in the first minute of more than 7, variables observed with low statistical significance (p > 0.05). However, the birth weight variable showed that newborns weighing over 2,501g developed more injuries (p = 0.0471), as well as those with an Apgar score in the fifth minute above 7 (p = 0.0001), as shown in Table 1.

**Table 1:** Distribution of newborns with some type of skin injury according to their characteristics at birth – Cascavel, PR, Brazil, 2023.

Characteristics	n	%	P-value
**Sex**			
Female	32	43.2	0.2955
Male	42	56.8	
**Birth weight (grams)**			
<1,000	10	13.5	0.0471
1,000 e <1,500	16	21.6	
1,500 e <2,500	22	29.7	
≥2,500	26	35.1	
**Gestational age (weeks)**			
<28	13	17.6	0.1462
28 e <32	9	12.2	
32 e <35	21	28.4	
35 e <37	12	16.2	
≥37	19	25.7	
**Apgar score 1** ^**st**^ **minute**			
0-3	25	33.8	0.1797
4-6	18	24.3	
7-10	31	41.9	
**Apgar score 5** ^**th**^ **minute**			
0-3	4	5.4	0.0001
4-6	13	17.6	
7-10	57	77.0	

Regarding the relationship between birth variables and the number of lesions, it was found that male newborns mainly developed more than one lesion. The appearance of three injuries was more prevalent among newborns with a birth weight of between 1,000g and 2,500g, with a gestational age of less than 28 weeks and between 32 and 35 weeks, and with an Apgar score in the first minute of less than 3 and in the fifth minute of more than 7. These data are shown in Table 2, all of which were not statistically significant.

**Table 2:** Distribution of newborns according to the number of injuries presented and characteristics at birth – Cascavel, PR, Brazil, 2023.

Characteristics	1 lesion		2 lesions		3 lesions		P-value
	n	%	n	%	n	%	
**Sex**							
Female	23	45.1	5	41.7	4	36.4	0.8668
Male	28	54.9	7	58.3	7	63.6	
**Birth weight (grams)**							
<1,000	5	9.8	3	25.0	2	18.2	0.0909
1,000 e <1,500	12	23.5	0	0.0	4	36.4	
1,500 e <2,500	15	29.4	3	25.0	4	36.4	
≥2,500	19	37.3	6	50.0	1	9.1	
**Gestational age (weeks)**							
<28	7	13.7	2	16.7	4	36.4	0.0802
28 e <32	8	15.7	1	8.3	0	0.0	
32 e <35	15	29.4	2	16.7	4	36.4	
35 e <37	8	15.7	1	8.3	3	27.3	
≥37	13	25.5	6	50.0	0	0.0	
**Apgar score 1** ^**st**^ **minute**							
0-3	16	31.4	3	25.0	6	54.5	0.4929
4-6	14	27.5	2	16.7	2	18.2	
7-10	21	41.2	7	58.3	3	27.3	
**Apgar score 5** ^**st**^ **minute**							
0-3	2	3.9	1	8.3	1	9.1	0.8540
4-6	8	15.7	2	16.7	3	27.3	
7-10	41	80.4	9	75.0	7	63.6	

Table 3 shows statistical significance in relation to the use of antibiotics (p = 0.0001) and parenteral nutrition (p = 0.0215). Newborns who used antibiotics had up to two lesions, while babies who did not use antibiotics had three lesions. Babies who used parenteral nutrition had up to two lesions, while those who didn’t, had three lesions.

**Table 3:** Distribution of newborns according to number of injuries, characteristics and interventions during hospitalization – Cascavel, PR, Brazil, 2023.

Characteristics	1 lesion		2 lesions		3 lesions		P-value
	n	%	n	%	n	%	
**Antibiotics**							
No	4	7.8	1	8.3	11	100.0	0.0001
Yes	47	92.2	11	91.7	0	0.0	
**Hemoglobin**							
< 11g/dl	6	11.8	3	25.0	3	27.3	0.2989
≥ 11g/dl	45	88.2	9	75.0	8	72.7	
**Total parenteral nutrition**							
No	32	62.7	10	83.3	3	27.3	0.0215
Yes	19	37.3	2	16.7	8	72.7	
**Phototherapy**							
No	20	39.2	5	41.7	5	45.5	0.9298
Yes	31	60.8	7	58.3	6	54.5	
**Length of stay**							
Up to 7 days	5	9.8	0	0.0	0	0.0	0.6450
8 -14 days	9	17.6	2	16.7	1	9.1	
15 - 30 days	12	23.5	4	33.3	2	18.2	
More than 30 days	25	49.0	6	50.0	8	72.7	
**NIPS** *							
<4	42	82.4	10	83.3	8	72.7	0.7736
≥4	9	17.6	2	16.7	3	27.3	

* *Neonatal Infant Pain Scale*.

Hemoglobin levels, phototherapy, hospitalization days and pain scores were not statistically significant. However, 82.7% of the newborns had hemoglobin levels of more than 11g/dl, and for those with one injury 88.2%, two 75% and three 72.7%. The majority who remained in phototherapy, with a pain score of less than 4 and a hospital stay of more than 30 days had one lesion.

In regards to the analysis of variance between the characteristics of the newborns and the number of skin lesions, no statistical significance was identified. It is important to note that the development of three injuries was predominant among newborns with a higher average birth weight, gestational age, Apgar score and length of hospital stay, although they took less time for the injuries to heal. For those who developed two lesions, lower birth weights, gestational ages and Apgar scores were identified, but they took longer to heal. Newborns with only one injury had a lower NSCS score and length of hospital stay, but a higher NIPS score, as shown in Table 4.

**Table 4:** Analysis of variance of birth weight, gestational age, 1st and 5th minute Apgar, skin condition score, pain score, healing days and hospitalization of hospitalized newborns distributed by number of lesions – Cascavel, PR, Brazil, 2023.

Characteristics	1 lesion	2 lesions	3 lesions	P-value
	Mean (SD)	Mean (SD)	Mean (SD)	
Birth weight	2091,51 (892,87)	1701,82 (705,26)	2311,25 (1147,25)	0.2716
Gestational age	33,27 (3,87)	31,27 (4,05)	34,42 (4,85)	0.1738
Apgar score 1^st^ minute	5,02 (2,87)	3,73 (2,80)	5,75 (3,19)	0.2440
Apgar score 5^th^ minute	7,33 (2,08)	6,64 (1,75)	7,58 (2,10)	0.5122
NSCS*	5,45 (0,64)	5,64 (0,81)	5,58 (0,67)	0.6426
NIPS**	1,65 (2,0)	1,55 (2,30)	1,42 (1,73)	0.9339
Healing (days)	33,33 (25,14)	50,73 (35,30)	10,22 (8,39)	0.5549
Hospitalization (days)	10,33 (11,63)	15,63 (19,94)	38,33 (23,23)	0.1450

* *Neonatal Skin Condition Score*;

** *Neonatal Infant Pain Scale*.

SD Standard Deviation.

Pearson’s correlation between the NSCS score and the birth and skin injury variables showed a weak correlation between the NSCS score and the 1st and 5th Apgar values (r = 0.309, p = 0.0008). The results showed that the higher the NSCS score, the lower the Apgar score at the 1st and 5th minute, hospitalization days, pain score (NIPS) and number of antibiotics used. However, statistically significant values were observed for the highest NSCS score and the greatest number of days for the injury to heal, the highest hemoglobin and hematocrit levels and the late start of the diet (Table 5).

**Table 5:** Pearson’s correlation between the Newborn Skin Conditions Score and birth weight, weight at the onset of the lesion, gestational age, 1^st^ and 5^th^ minute Apgar scores, healing time and hospitalization, pain score, hemoglobin, hematocrit, number of antibiotics used and start of diet – Cascavel, PR, Brazil, 2023.

Variables	N	R*	CI (95%)	Valor p
Birth weight	114	–0.030	(–0.15; 0.21)	0.7305
Weight at onset of lesion	114	–0.023	(0.21; 0.16)	0.8058
Gestational age	114	–0.011	(–0.19; 0.17)	0.9036
Apgar 1^st^ minute	114	–0.243	(–0.41; 0.06)	0.0089
Apgar score 5^th^ minute	114	–0.309	(–0.47; –0.13)	0.0008
Healing (days)	87	0.106	(–0.11; 0.31)	0.3274
Hospitalization (days)	114	–0.223	(–0.39; –0.04)	0.0168
NIPS**	114	–0.239	(–0.41; –0.06)	0.0104
Hemoglobin	113	0.198	(0.01; 0.37)	0.0357
Hematocrit	113	0.195	(0.01; 0.37)	0.0388
Amount of ATB***	114	–0.218	(–0.39; –0.04)	0.0193
Start of diet (days)	109	0.196	(0.01; 0.37)	0.0403

* Pearson correlation;

** *Neonatal Infant Pain Scale*;

*** Antibiotics. CI Confidence Interval.

## DISCUSSION

The frequency of skin injuries among hospitalized newborns in this study was considered low to moderate when compared to other studies^(5,6)^, however, more than half of these babies had more than one type of lesion, with dermatitis and pressure injuries being the most common.

A Brazilian study highlighted that the rate of skin injuries reaches 40% among hospitalized newborns, with males being the most affected. The most frequent injuries in this study were related to peripheral access and nasal septum injuries due to the use of non-invasive mechanical ventilation^(12)^. In Italy, the injury rate can reach 70% and neonatal toxic erythema was the most common type found^(6)^.

Premature newborns have an immature epidermal layer and greater trans-epidermal water loss. In addition to these characteristics, pressure injuries can occur due to a lack of oxygen and nutrients, as well as the prolonged use of medical devices, lack of decubitus changes and skin protection during the use of devices^(12,13)^. It is important to note that pressure injuries were quite common in this study.

Dermatitis, which is quite evident, has been considered common among hospitalized newborns, due to the anatomical and physiological particularities of the baby, the use of chlorhexidine, the humidity caused by diapers and the way perianal hygiene is performed^(13)^.

With regard to characterizing the babies who developed skin lesions, it was found that the majority were male, with a gestational age between 32 and 34 weeks and six days and an Apgar score in the first minute of more than 7. Newborns with adequate birth weight developed more than one type of skin lesion, as did those with an Apgar score of more than 7 in the fifth minute. When the score is low (<7), the baby can suffer circulatory, respiratory and neurological damage, factors that directly affect the skin, especially in premature babies, since, like other organs and systems, the skin is still developing^(14)^. With regard to males, although there is a study^(12)^ that corroborates this result, there is no evidence linking them to skin lesions.

The occurrence of various types of injuries was higher for extreme and moderate preterm babies, with the latter mainly developing perianal dermatitis.

Newborns have different characteristics at each stage of development. The stratum corneum becomes functionally mature between 32 and 34 weeks of gestational age, while babies born at less than 24 weeks do not yet have this layer. It has to be considered that the intrauterine environment is moist, warm and sterile, suitable for development. After birth, babies have to adapt to a hostile, cold, dry environment full of microorganisms, causing trans-epidermal water loss and susceptibility to epidermal infections and lesions^(5)^.

Most of the newborns who developed injuries were of adequate weight, i.e. over 2,500g; however, babies with a lower weight developed a greater number of lesions. Global study on skin injuries in neonatal units conducted by researchers from several countries involving mainly extremely premature and low birth weight babies, showed that 39% developed diaper dermatitis and 38% injuries related to the use of medical devices. An important factor observed by the researchers was the difference in these percentages in units organized with specific skin care protocols^(15)^.

An additional highlighted result was the relationship between the use of antibiotics and parenteral nutrition and the appearance of skin lesions. The newborns who used more than one type of antibiotic had one and two lesions, while the babies who didn’t, who were fewer in number, had three lesions. The use of antibiotics corroborates the decrease in intestinal microbiota, making babies more susceptible to the inflammatory process and potentially exposed to colonization by pathogenic bacteria, factors that interfere with skin development^(16)^. A study on dermatitis associated with fecal incontinence showed that the use of antibiotics can influence the appearance of perianal lesions^(16)^.

It was found that newborns who did not use parenteral nutrition had a higher chance of developing skin lesions, but when comparing the number of lesions, the babies who did use parenteral nutrition had a higher number, a fact possibly justified by the length of hospitalization. The scientific literature describes that prematurity leads to nutritional deficiency, justified by the interrupted supply, which can lead to malnutrition and with it, deficits in growth and maturation, including of the skin. Parenteral nutrition provides support that increases quality of life, stimulates intestinal maturation and reduces nutrient loss^(17)^.

Regarding hemoglobin levels, it was found that babies with values higher than 11g/dl developed skin lesions, i.e. having anemia or not did not influence the appearance of lesions. A study on the development of pressure injuries showed that biochemical markers are directly related to the appearance of injuries, so monitoring hemoglobin levels is important and interferes with recovery, nutrition and oxygenation of the skin^(18)^.

Although the use of phototherapy has failed to show to be related to the appearance and number of lesions, it should be noted that this is not just a risk factor, but a factor that can make treatment more difficult when the injury already exists.

Hyperbilirubinemia affects between 60 and 70% of full-term newborns and between 80 and 90% of premature infants, making phototherapy treatment necessary. This is a non-invasive therapy that acts directly on the skin, and the larger the area exposed, the more effective it is. Care measures include a distance of between 30cm and 50cm between the lamp and the baby, depending on the type of lamp; eye protection; temperature monitoring; weight control; control of mucosal moisture and skin turgor; changes in position; not using topical products or emollients. These precautions are important to prevent retinal damage, burns, dehydration, temperature changes, rashes and erythema during treatment^(19,20)^.

One study showed that the use of emollients on dry, flaking or cracked skin is important to prevent trans-epidermal water loss, remove adhesives and perform body hygiene with barrier creams^(5)^. However, this care should be postponed when the newborn is undergoing phototherapy, as it favors the development of lesions.

The majority of newborns with skin injuries did not have a pain score on the NIPS scale, but premature infants do not have the ability to modulate, inhibit or reduce pain. They perceive pain more intensely than adults and it lasts for a while after the end of stressful stimuli. Preventing skin injuries that cause pain is crucial for good development^(21)^.

A study on pain assessment practices highlighted that the use of scales helps in the early detection and prevention of pain relief. When faced with a stressful situation that causes pain, the study suggested the use of hammocks for positioning, mobilization in the lateral and ventral decubitus position, favoring alignment and the flexor position, facilitated touch, the use of 25% sucrose, non-nutritive sucking, the kangaroo position, breastfeeding, reducing light, noise and stimuli^(21)^.

With regard to length of hospital stay, a period of more than 30 days was found for those with three lesions. Studies show that skin injuries are responsible for increasing the length of stay in neonatal units by around 37% to 40%^(22,23)^.

Pearson’s correlation showed worrying results for the highest NSCS score and the greatest number of days for the injury to heal and late start of the diet. It is known that gastrointestinal immaturity hinders the supply and use of nutrients via the enteral route. However, early initiation of a trophic diet stimulates the motility of this system and reduces adverse events. Breast milk is always the main choice, as it favors development, reduces necrotizing enterocolitis, sepsis and other complications of prematurity^(24)^.

Moreover, skin injuries are considered a gateway for various microorganisms, so the longer the non-integrated skin is exposed, the greater the chances of adverse events occurring and the longer the hospital stay^(25)^.

Evaluating the skin of newborn babies is considered complex, and when they are extremely premature, their thin, gelatinous skin makes injuries less noticeable, especially when they are plethoric. In this way, skin care for these babies becomes important in order to protect their integrity and their thermoregulatory function, directly influencing quality of care indicators, since the presence of one or more skin injuries contributes to an increase in infection rates and other complications during hospitalization^(1,26)^. The reduced subcutaneous structure of the skin of premature and low birth weight newborns is often not differentiated from that of other infants in terms of care, contributing to increased susceptibility to injuries^(26)^.

Because of the presence of skin injuries among hospitalized newborns, the nursing team plays an important role in preventing, maintaining and restructuring the skin. This triad of care includes changing the position of the baby, maintaining temperature, careful hygiene, the use of emollients, care with the administration of medication, dressings, protection against friction from tubes, probes and other equipment necessary for the baby’s recovery^(27)^. It is up to nurses to draw up protocols, as well as maintaining these practices in the daily routine of care in a neonatal unit.

### Limitations of the Study

A limitation of the study was the incompleteness of the nursing team’s notes in the electronic medical records, since changes in skin conditions were identified by the NSCS, but were not described in the nursing evolution. Another limiting factor was the completion of the NIPS scale, resulting in the omission of information about the pain of newborns with skin lesions. It is therefore recommended that the nursing team become aware of the importance of recording information about hospitalized newborns in a complete and appropriate way, given the urgency of ensuring a care practice based on their real needs, contributing to the prevention and treatment of skin lesions.

### Advances for the Nursing Field

The study was important for the nursing field because it provided knowledge about the factors and characteristics that can increase the chances of a newborn developing skin lesions, as well as the need to know the anatomical and physiological particularities of the skin of premature infants, so that nurses can identify the risks early and intervene in a timely manner, aiming to reduce their occurrence, quality of care, shorter hospitalization time, morbidity and mortality, hospital costs and suffering for newborns and their families.

## CONCLUSION

The study showed a low to moderate frequency of skin injuries among hospitalized newborns, more than half of them presenting more than one type of lesion, predominantly dermatitis and pressure injuries. Most of the babies had an adequate birth weight and Apgar scores >7, however, the greatest diversity of injuries affected extreme and moderate premature infants. Newborns who did not use parenteral nutrition were more likely to develop lesions; being under phototherapy, the presence of anemia and the use of the highest number of antibiotics did not influence the appearance of lesions. Babies with three injuries remained in hospital for more than 30 days.

Based on this text, some implications for the nursing service can be identified: the nursing team must implement skin injury prevention practices in newborns, developed and followed rigorously, especially in the neonatal ICU. It is also crucial to carry out continuous assessment of the skin condition of hospitalized newborns, allowing for early interventions, as well as developing protocols and continuing education for multi-professional staff with early screening of risk factors. Given the relationship between the number of injuries and healing time, the nursing team must be prepared to manage complications arising from multiple lesions, including a specific care plan for each newborn with two or more injuries.

Therefore, it is understood that there is a need to improve multi-professional care with regard to the particularities of hospitalized babies, which include the practices and conduct carried out on a daily basis to prevent and maintain skin integrity, given the importance of quality and humanization of care, patient safety, reducing hospitalization time and hospital costs.

Furthermore, the use of the NSCS proved to be important and should be used as an ally in nursing care, as it helps in the early identification of skin lesions, as well as their prevention. It is therefore suggested that further studies should be carried out on this subject in order to expand knowledge and improve care protocols in neonatology.
