# Supplementary material for: Skin injuries in newborns hospitalized in neonatal intensive care: a cross-sectional study
Source: Rev Esc Enferm USP. 2024 Aug 2;58:e20240058. doi: 10.1590/1980-220X-REEUSP-2024-0058en (PMC11321824; doi:10.1590/1980-220X-REEUSP-2024-0058en)
Supplement: Translation

# Translation: Lesão de pele em recém-nascidos hospitalizados em terapia intensiva neonatal: estudo seccional*

**DOI:** 10.1590/1980-220X-REEUSP-2024-0058pt

**Authors:** Carolina Tenfen, Grasiely Masotti Scalabrin Barreto, Neide Martins Moreira, Helder Ferreira, Adriana Zilly, Rosane Meire Munhak da Silva

**Affiliations:** 1 Hospital Universitário do Oeste do Paraná, Programa de Residência Multiprofissional em Neonatologia. Cascavel, PR, Brasil.; 2 Universidade Estadual do Oeste do Paraná, Centro de Educação, Letras e Saúde, Foz do Iguaçu, PR, Brasil.; 3 Universidade Estadual do Oeste do Paraná, Centro de Educação, Letras e Saúde, Programa de Residência Multiprofissional em Neonatologia, Cascavel, PR, Brasil.

**Keywords:** Ferimentos e Lesões, Pele, Recém-Nascido, Unidades de Terapia Intensiva Neonatal, Enfermagem Neonatal

## Abstract

**Objetivo:**

Analisar as lesões de pele de recém-nascidos hospitalizados e identificar fatores relacionados ao número de lesões.

**Método:**

Estudo epidemiológico seccional, realizado no período de um ano, em Unidade de Terapia Intensiva Neonatal do Oeste do Paraná. Foram incluídos 74 recém-nascidos com escore ≥5 na Escala de Condição da Pele do Recém-Nascido. Análise de dados por teste qui-quadrado e Pearson (p < 0,05).

**Resultados:**

A frequência foi de 25,4%, 59,4% apresentaram mais de uma lesão, principalmente dermatites e lesão por pressão. Características do nascimento não se relacionaram ao número de lesões. Não usar antibióticos e nutrição parenteral, hemoglobina >11g/dl, fototerapia, escore de dor <4 e hospitalização >30 dias foram relacionados ao número de lesões. Presença de duas lesões levou maior tempo de cicatrização e três, de hospitalização. Maior escore na Escala de Condição da Pele se relacionou ao tempo de cicatrização e início tardio da dieta.

**Conclusão:**

A lesão de pele mostrou-se pouco frequente entre os recém-nascidos, mesmo assim, é preciso aprimorar as práticas para prevenir e manter a integridade da pele.

## INTRODUÇÃO

A pele é o maior órgão do ser humano e é responsável por funções importantes no organismo. O recém-nascido, em especial o prematuro, possui características peculiares, sendo a carência estrutural inversamente proporcional à idade gestacional, o que leva a problemas relacionados com desidratação, instabilidade térmica, desequilíbrios eletrolíticos, atraso na maturação, maior suscetibilidade ao desenvolvimento de traumas e de lesões^(1)^. A pele do bebê prematuro é 30% mais fina e não está completamente desenvolvida até 34 semanas de idade gestacional, e essas características a tornam mais frágil, causando irritações, alergias e infecções mais facilmente^(2)^.

Muitos recém-nascidos prematuros e de baixo peso requerem cuidados hospitalares para suprir suas necessidades de oxigenação, circulação e do metabolismo. Para isso, intervenções invasivas e não-invasivas são necessárias, as quais por um lado garantem a sobrevida desses bebês, mas por outro causam inúmeros eventos adversos. Entre esses eventos causados pelos cuidados assistenciais no começo da vida, encontram-se as lesões de pele^(3)^.

Quando o recém-nascido é hospitalizado, por muitas vezes é submetido a realização de procedimentos que necessitam de assepsia e antissepsia, banhos (higienização), coleta de exames laboratoriais e curativos, além de utilizar diversos dispositivos, como: tubos orotraqueais, cânulas nasais (prongas), sondas, cateteres, sensores, fraldas e fixações, que, em contato direto com a pele, a torna suscetível ao desenvolvimento de lesões^(4)^.

A prevalência de lesões de pele em recém-nascido é alta, cerca de 80% desenvolvem algum tipo de lesão de pele no primeiro mês de vida, 25% de todos os prematuros e bebês de baixo peso apresentam o diagnóstico de sepse precoce, apresentando a pele não integra como a principal porta de entrada^(5)^. Uma pesquisa realizada na Itália revelou que 70% dos bebês hospitalizados em terapia intensiva, prematuros e a termo, apresentaram pelo menos uma alteração na pele^(6)^.

Um dos desafios para a equipe multiprofissional é garantir segurança ao paciente, sobretudo em Unidades de Terapia Intensiva (UTI), uma vez que, comportam pacientes com maior gravidade e com isso maior ocorrência de eventos adversos, pela instabilidade clínica, alto número de intervenções e dispositivos invasivos e não invasivos^(7)^.

A prevenção e os cuidados com a pele de recém-nascidos se encontram amplamente relacionados a assistência de enfermagem, por considerar que este profissional mantém o contato direto com os bebês para realizar suas atribuições diárias, necessárias para a sobrevivência e recuperação de sua saúde. Para tanto, sua prática assistencial precisa ser aprimorada a cada dia, lançando mão de ferramentas como a Sistematização da Assistência de Enfermagem para nortear o cuidado a ser realizado pela equipe de enfermagem, proporcionando uma assistência humanizada e de qualidade^(7,8)^.

A inspeção diária da pele, presente nas práticas de cuidados do enfermeiro, pode fortalecer a preservação e proteção da pele do recém-nascido que necessita do uso de aparatos tecnológicos. Para isso, o enfermeiro deve buscar conhecimento e práticas baseadas em evidências que auxiliam na sistematização do cuidado, contribuindo com a segurança e a qualidade, e principalmente para minimizar o desconforto, a dor e o tempo de hospitalização^(9)^. Para a avaliação da integridade da pele de recém-nascidos, a literatura indica o uso de escalas, a exemplo, a Escala de Condição da Pele do Recém-Nascido (ECPRN), a qual propicia a detecção precoce de lesões, padroniza a avaliação pelos profissionais de saúde e individualiza o cuidado^(10)^.

Por considerar a relevância da manutenção da integridade da pele de recém-nascidos para sua plena recuperação, essa pesquisa teve como questionamentos: Qual a incidência e quais as principais lesões de pele em recém-nascidos hospitalizados em terapia intensiva neonatal? Quais fatores potencializam a sua recorrência? Desse modo, a pesquisa teve como objetivo analisar as lesões de pele de recém-nascidos hospitalizados e identificar fatores relacionados ao número de lesões.

## MÉTODO

### Desenho do Estudo

Estudo epidemiológico seccional, que utilizou as diretrizes para estudos observacionais do *Strengthening the Reporting of Observational Studies* (STROBE).

### Local do Estudo

Foi desenvolvido em uma UTI Neonatal de um hospital universitário da região oeste do estado do Paraná. A referida UTI Neonatal dispõe de dez leitos estruturados para o atendimento de recém-nascidos gravemente enfermos, sendo que, destes leitos, um está organizado para casos que necessitem de isolamento, independentemente da origem. Grande parte dos recém-nascidos admitidos na unidade são procedentes do Centro Obstétrico e Unidade de Cuidados Intermediários Neonatal da própria instituição, mas também admite recém-nascidos dos demais municípios pertencentes a décima Regional de Saúde do Estado do Paraná.

### Critérios de Seleção

Foram incluídos no estudo os recém-nascidos hospitalizados na UTI Neonatal, independentemente da idade gestacional e/ou diagnóstico de internação, que em algum momento apresentaram um escore igual ou superior a 5 na ECPRN. Ressalta-se que a referida escala é realizada diariamente pelo enfermeiro da unidade. Foram excluídos os recém-nascidos que necessitaram de transferência para outra UTI Neonatal sem o término do tratamento da lesão de pele e aqueles que permaneceram hospitalizados após o término do período de coleta de dados.

### A Ecprn

A ECPRN teve sua adaptação transcultural e validação para uso no Brasil em 2012, a partir da escala *Neonatal Skin Condition Score* (NSCS), sendo a única já validada nos Estados Unidos da América (EUA) para uso na população neonatal. Essa, é dividida em três tópicos a serem avaliados: secura, eritema e ruptura/lesão, dentro desses, subtópicos, em secura: é atribuído valor 1 para pele normal, nenhum sinal de pele seca, 2 para pele seca, descamação visível e 3 para pele muito seca, rachaduras/ fissuras; em eritema: é atribuído valor 1 para não há evidências de eritema, 2 para eritema visível, <50% da superfície corporal e 3 para eritema visível, ≥50% da superfície corporal; em ruptura/ lesão: é atribuído valor 1 para nenhuma visível, 2 para pequena em áreas localizadas e 3 para extensa. A pontuação mínima e ideal é 3 e o pior resultado é 9^(10)^.

### Coleta de Dados

A coleta de dados foi realizada durante o período de um ano, com início em outubro de 2022 e finalização em outubro de 2023, por meio de busca em prontuários eletrônicos, por uma enfermeira, residente na unidade de neonatologia. A coleta foi iniciada após a implantação da ECPRN no sistema Tasy^®^ e treinamento da equipe de enfermeiros da unidade, pela coletadora, e posteriormente, a partir do momento que o primeiro recém-nascido recebeu escore igual ou superior a 5 na ECPRN foi dado início a coleta.

Foi utilizado um instrumento estruturado, organizado pela pesquisadora responsável, a qual tem experiência na área e em pesquisas em neonatologia. Foram incluídas as seguintes variáveis: i) *Dados do nascimento*: data do nascimento, sexo, peso ao nascer, idade gestacional, escores de Apgar no primeiro e quinto minuto; ii) *Dados da hospitalização*: data da hospitalização, complicações, dias de vida em que foi iniciado a dieta, utilização de nutrição parenteral e desfecho: alta ou óbito; iii) *Dados iniciais da lesão*: dias de vida no aparecimento da lesão, peso, tipo de lesão, escore de ECPRN, infecção (presente ou ausente), antibióticos utilizados, exames laboratoriais (hemoglobina e hematócrito), origem da lesão, permanência em fototerapia, escore na *Neonatal Infant Pain Scale* (NIPS)^(11)^; iiii) *Evolução da lesão*: se houve piora da lesão, recorrência da lesão (sim ou não).

### Análise e Tratamento dos Dados

Os dados foram organizados em planilhas no Excel^®^, por dupla digitação independente. Para análise dos dados, as variáveis estudadas foram organizadas em tabelas de dupla entrada e realizado o cálculo de frequências absolutas e relativas. Buscou-se na análise identificar e relacionar aspectos do nascimento, evolução e o aparecimento de novas lesões de pele no recém-nascido. Foi utilizado o teste de qui-quadrado com nível significância de 5% para verificar essa associação. Também foi realizada a análise de correlação de Pearson entre o escore da ECPRN e as variáveis de nascimento e da lesão de pele, utilizando-se o programa XLStat2014^®^.

### Aspectos Éticos

A pesquisa foi aprovada pelo Comitê de Ética em Pesquisa da Universidade, parecer nº 5.656.829 no ano de 2022 e obedeceu à Resolução 466/2012 do Conselho Nacional de Saúde que regulamenta a pesquisa com seres humanos no Brasil. Foi solicitado o consentimento dos pais responsáveis pelo recém-nascido, sendo que, após explicar os objetivos da pesquisa e sua anuência, foi assinado o Termo de Consentimento Livre e Esclarecido, em duas vias. A confidencialidade das informações foi assegurada por meio da codificação dos recém-nascidos.

## RESULTADOS

No período de estudo, 291 recém-nascidos foram hospitalizados na UTI neonatal e 25,4% (n = 74) apresentaram algum tipo de lesão de pele. Desses, 59,4% (n = 44) apresentaram mais de um tipo de lesão, sendo que as principais identificadas foram dermatites e lesão por pressão.

A maioria dos recém-nascidos com lesão de pele eram do sexo masculino, com idade gestacional entre 32 a 34 semanas e seis dias e escore de Apgar no primeiro minuto superior a 7, variáveis observadas com fraca significância estatística (p > 0,05). Entretanto, a variável peso ao nascer mostrou que recém-nascidos com peso acima de 2.501g desenvolveram mais lesões (p = 0,0471), bem como, aqueles que apresentaram escore de Apgar no quinto minuto acima de 7 (p = 0,0001), como mostra a Tabela 1.

**Tabela 1:** Distribuição dos recém-nascidos que apresentaram algum tipo de lesão de pele segundo suas características ao nascer – Cascavel, PR, Brasil, 2023.

Características	N	%	Valor-p
**Sexo**			
Feminino	32	43,2	0,2955
Masculino	42	56,8	
**Peso ao nascer (gramas)**			
<1.000	10	13,5	0,0471
1.000 e <1.500	16	21,6	
1.500 e <2.500	22	29,7	
≥2.500	26	35,1	
**Idade gestacional (semanas)**			
<28	13	17,6	0,1462
28 e <32	9	12,2	
32 e <35	21	28,4	
35 e <37	12	16,2	
≥37	19	25,7	
**Escore de Apgar 1º minuto**			
0-3	25	33,8	0,1797
4-6	18	24,3	
7-10	31	41,9	
**Escore de Apgar 5º minuto**			
0-3	4	5,4	0,0001
4-6	13	17,6	
7-10	57	77,0	

Com respeito a relação entre as variáveis de nascimento e o número de lesões, verificou-se que os recém-nascidos do sexo masculino desenvolveram principalmente mais de uma lesão. O aparecimento de três lesões foi mais predominante entre recém-nascidos com peso ao nascer entre 1.000g e 2.500g, com idade gestacional inferior a 28 semanas e entre 32 e 35 semanas, e com escore de Apgar no primeiro minuto inferior a 3 e no quinto minuto superior a 7. Esses dados são apresentados na Tabela 2, todos os quais não mostraram relevância estatística.

**Tabela 2:** Distribuição de recém-nascidos segundo o número de lesões apresentadas e características ao nascimento – Cascavel, PR, Brasil, 2023.

Características	1 lesão		2 lesões		3 lesões		Valor-p
	n	%	n	%	n	%	
**Sexo**							
Feminino	23	45,1	5	41,7	4	36,4	0,8668
Masculino	28	54,9	7	58,3	7	63,6	
**Peso ao nascer (gramas)**							
<1.000	5	9,8	3	25,0	2	18,2	0,0909
1.000 e <1.500	12	23,5	0	0,0	4	36,4	
1.500 e <2.500	15	29,4	3	25,0	4	36,4	
≥2.500	19	37,3	6	50,0	1	9,1	
**Idade gestacional (semanas)**							
<28	7	13,7	2	16,7	4	36,4	0,0802
28 e <32	8	15,7	1	8,3	0	0,0	
32 e <35	15	29,4	2	16,7	4	36,4	
35 e <37	8	15,7	1	8,3	3	27,3	
≥37	13	25,5	6	50,0	0	0,0	
**Escores de Apgar 1º**							
0-3	16	31,4	3	25,0	6	54,5	0,4929
4-6	14	27,5	2	16,7	2	18,2	
7-10	21	41,2	7	58,3	3	27,3	
**Escores de Apgar 5º**							
0-3	2	3,9	1	8,3	1	9,1	0,8540
4-6	8	15,7	2	16,7	3	27,3	
7-10	41	80,4	9	75,0	7	63,6	

Na Tabela 3 pode-se observar significância estatística em relação ao uso de antibióticos (p = 0,0001) e nutrição parenteral (p = 0,0215). Os recém-nascidos que utilizaram antibióticos apresentaram até duas lesões, enquanto os bebês que não utilizaram, três lesões. Os bebês que utilizaram nutrição parenteral apresentaram até duas lesões, enquanto os que não utilizaram, três lesões.

**Tabela 3:** Distribuição de recém-nascidos segundo o número de lesões apresentadas, características e intervenções na hospitalização – Cascavel, PR, Brasil, 2023.

Características	1 lesão		2 lesões		3 lesões		Valor-p
	n	%	n	%	n	%	
**Antibióticos**							
Não	4	7,8	1	8,3	11	100,0	0,0001
Sim	47	92,2	11	91,7	0	0,0	
**Hemoglobina**							
< 11g/dl	6	11,8	3	25,0	3	27,3	0,2989
≥ 11g/dl	45	88,2	9	75,0	8	72,7	
**Nutrição Parenteral Total**							
Não	32	62,7	10	83,3	3	27,3	0,0215
Sim	19	37,3	2	16,7	8	72,7	
**Fototerapia**							
Não	20	39,2	5	41,7	5	45,5	0,9298
Sim	31	60,8	7	58,3	6	54,5	
**Dias de internação**							
Até 7 dias	5	9,8	0	0,0	0	0,0	0,6450
8 a 14 dias	9	17,6	2	16,7	1	9,1	
15 a 30 dias	12	23,5	4	33,3	2	18,2	
Mais de 30 dias	25	49,0	6	50,0	8	72,7	
**NIPS** *							
<4	42	82,4	10	83,3	8	72,7	0,7736
≥4	9	17,6	2	16,7	3	27,3	

* *Neonatal Infant Pain Scale*.

Os níveis de hemoglobina, fototerapia, dias de internação e escore de dor não obtiveram significância estatística. Contudo, 82,7% dos recém-nascidos apresentaram níveis de hemoglobina maior de 11g/dl, sendo que, para aqueles com uma lesão 88,2%, duas 75% e três 72,7%. A maioria que permaneceu em fototerapia, com escore de dor menor que 4 e tempo de hospitalização superior a 30 dias apresentaram uma lesão.

Com respeito a análise da variância das características dos recém-nascidos com o número de lesão de pele, não foi identificada relevância estatística. Importante destacar que o desenvolvimento de três lesões foi predominante entre recém-nascidos com maior média de peso, idade gestacional, escore de Apgar e tempo de hospitalização, todavia, levaram menor tempo de cicatrização das lesões. Para os que desenvolveram duas lesões, foram identificados menor peso ao nascer, idade gestacional e escores de Apgar, mas levaram maior tempo para cicatrização. Recém-nascidos com apenas uma lesão apresentaram menor escore na ECPRN e tempo de hospitalização, apresentando, contudo, maior escore no NIPS, conforme demonstra a Tabela 4.

**Tabela 4:** Análise da variância do peso ao nascer, idade gestacional, Apgar de 1º e 5º minuto, escore de condições da pele, escore de dor, dias de cicatrização e hospitalização de recém-nascidos hospitalizados distribuídos por número de lesões – Cascavel, PR, Brasil, 2023.

Características	1 lesão	2 lesões	3 lesões	Valor-p
	Média (DP)	Média (DP)	Média (DP)	
Peso ao nascer	2091,51 (892,87)	1701,82 (705,26)	2311,25 (1147,25)	0,2716
Idade gestacional	33,27 (3,87)	31,27 (4,05)	34,42 (4,85)	0,1738
Apgar 1º minuto	5,02 (2,87)	3,73 (2,80)	5,75 (3,19)	0,2440
Apgar 5º minuto	7,33 (2,08)	6,64 (1,75)	7,58 (2,10)	0,5122
ECPRN*	5,45 (0,64)	5,64 (0,81)	5,58 (0,67)	0,6426
NIPS**	1,65 (2,0)	1,55 (2,30)	1,42 (1,73)	0,9339
Cicatrização (dias)	33,33 (25,14)	50,73 (35,30)	10,22 (8,39)	0,5549
Hospitalização (dias)	10,33 (11,63)	15,63 (19,94)	38,33 (23,23)	0,1450

* Escala de Condição da Pele do Recém-Nascido;

** *Neonatal Infant Pain Scale*.

Ao calcular a correlação de Pearson entre o escore da ECPRN e as variáveis de nascimento e da lesão de pele, observou-se fraca correlação do escore da ECPRN e os valores de Apgar 1º e 5º (r = 0,309, p = 0,0008). Os resultados mostraram que quanto maior o escore da ECPRN, menor o Apgar no primeiro e quinto minuto, dias de hospitalização, escore de dor (NIPS) e número de antibióticos utilizados. Contudo, observou-se valores com significância estatística para o maior escore da ECPRN e o maior número de dias para cicatrização da lesão, maior nível de hemoglobina, hematócrito e início tardio da dieta (Tabela 5).

**Tabela 5:** Correlação de Pearson entre Escore de Condições da Pele do Recém-nascido e o peso ao nascer, peso no aparecimento da lesão, idade gestacional, Apgar de 1º e 5º minuto, tempo de cicatrização e hospitalização, escore de dor, hemoglobina, hematócrito, número de antibióticos usados e início da dieta – Cascavel, PR, Brasil, 2023.

Variáveis	N	R*	IC (95%)	Valor p
Peso ao nascer	114	–0,030	(–0,15; 0,21)	0,7305
Peso no aparecimento da lesão	114	–0,023	(0,21; 0,16)	0,8058
Idade Gestacional	114	–0,011	(–0,19; 0,17)	0,9036
Apgar 1º minuto	114	–0,243	(–0,41; 0,06)	0,0089
Apgar 5º minuto	114	–0,309	(–0,47; –0,13)	0,0008
Cicatrização (dias)	87	0,106	(–0,11; 0,31)	0,3274
Hospitalização (dias)	114	–0,223	(–0,39; –0,04)	0,0168
NIPS**	114	–0,239	(–0,41; –0,06)	0,0104
Hemoglobina	113	0,198	(0,01; 0,37)	0,0357
Hematócrito	113	0,195	(0,01; 0,37)	0,0388
Quantidade de ATB***	114	–0,218	(–0,39; –0,04)	0,0193
Início da dieta (dias)	109	0,196	(0,01; 0,37)	0,0403

* Correlação de Pearson;

** *Neonatal Infant Pain Scale*;

*** Antibióticos.

## DISCUSSÃO

A frequência de lesão de pele entre os recém-nascidos hospitalizados dessa investigação foi considerada baixa a moderada, ao ser comparada com outros estudos^(5,6)^, contudo, mais da metade desses bebês apresentaram mais de um tipo de lesão, sendo que, as dermatites e a lesão por pressão foram as de maior ocorrência.

Um estudo brasileiro destacou que a taxa de lesões de pele chega a 40% entre recém-nascidos hospitalizados, sendo o sexo masculino o mais acometido. As lesões mais frequentes nesse estudo foram relacionadas aos acessos periféricos e as de septo nasal pelo uso de ventilação mecânica não invasiva^(12)^. Na Itália, a taxa de lesão pode chegar a 70% e o eritema tóxico neonatal foi o tipo mais encontrado^(6)^.

Os recém-nascidos prematuros apresentam imaturidade da camada epidérmica e maior perda de água transepidérmica. Somado a essas características, as lesões por pressão podem ocorrer pela falta de oxigênio e nutrientes, e também pelo uso prolongado de dispositivos médicos, falta de mudança de decúbito e de proteção da pele durante a utilização dos dispositivos^(12,13)^. Importante destacar que nesse estudo as lesões por pressão foram bastante frequentes.

A dermatite, bastante evidente, tem sido considerada comum entre recém-nascidos hospitalizados, tanto pelas particularidades anatômicas e fisiológicas do bebê, como pelo uso de clorexidinas, pela umidade ocasionada por fraldas e a maneira como é realizada a higiene perianal^(13)^.

Com respeito a caracterização dos bebês que evoluíram com lesão de pele, identificou-se que a maioria foi do sexo masculino, com idade gestacional entre 32 e 34 semanas e seis dias e escore de Apgar no primeiro minuto superior a 7. Recém-nascidos com peso adequado desenvolveram mais de um tipo de lesão de pele, bem como, os que apresentaram escore de Apgar no quinto minuto superior a 7. Quando o escore é baixo (<7), esse bebê pode sofrer prejuízos circulatórios, respiratórios e neurológicos, fatores que interferem diretamente na pele, em especial do prematuro, visto que, como os demais órgãos e sistemas, a pele ainda está em desenvolvimento^(14)^. Com respeito ao sexo masculino, embora tenha estudo^(12)^ que corrobora com esse resultado, não há evidências que o relacionem com a lesão de pele.

A ocorrência de vários tipos de lesão foi maior para bebês prematuros extremos e moderados, sendo que estes últimos desenvolveram sobretudo a dermatite perianal.

Os recém-nascidos apresentam características distintas em cada fase do desenvolvimento. O estrato córneo torna-se funcionalmente maduro entre 32 a 34 semanas de idade gestacional, já bebês que nascem com idade inferior a 24 semanas, ainda não apresentam essa camada. É preciso considerar que o ambiente intrauterino é úmido, aquecido e estéril, adequado ao desenvolvimento, enquanto após o nascimento, os bebês precisam adaptar-se à um ambiente hostil, frio, seco e repleto de microrganismos, causando perda de água transepidérmica e susceptibilidade às infecções epidérmicas e lesões^(5)^.

A maioria dos recém-nascidos que desenvolveu lesões tinham peso adequado, ou seja, acima de 2.500g, entretanto, os bebês com peso inferior desenvolveram um número maior de lesões. Estudo global sobre lesões cutâneas em unidades neonatais conduzido por pesquisadores de vários países envolvendo principalmente bebês prematuros extremos e com baixo peso ao nascer, mostrou que 39% desenvolveram dermatite de fraldas e 38% lesões relacionadas ao uso de dispositivos médicos. Fator importante observado pelos pesquisadores foi a diferença desses percentuais em unidades organizadas com protocolos específicos para os cuidados com a pele^(15)^.

Resultado também destacado foi a relação do uso de antibióticos e da nutrição parenteral com o aparecimento de lesão de pele. Os recém-nascidos que utilizaram mais de um tipo de antibiótico apresentaram uma e duas lesões, já os bebês que não utilizaram, que foram em número menor, apresentaram três lesões. O uso de antibióticos corrobora com a diminuição da microbiota intestinal, tornando os bebês mais suscetíveis ao processo inflamatório e potencialmente expostos a colonização por bactérias patogênicas, fatores que interferem no desenvolvimento da pele^(16)^. Um estudo sobre dermatite associada a incontinência fecal, mostrou que a utilização de antibióticos pode influenciar o aparecimento de lesões perianais^(16)^.

Verificou-se que os recém-nascidos que não utilizaram nutrição parenteral apresentaram uma chance maior de evoluir com lesões de pele, porém, para os bebês que utilizaram essa nutrição, ao comparar o número de lesões, apresentaram maior número, fato justificado possivelmente pelo tempo de hospitalização. A literatura científica descreve que a prematuridade leva a carência nutricional, justificada pela oferta interrompida, podendo levar a desnutrição e com ela, déficit no crescimento e amadurecimento, inclusive da pele. A nutrição parenteral fornece subsídios que aumentam a qualidade de vida, estimulam a maturação do intestino e reduz a perda de nutrientes^(17)^.

Sobre os níveis de hemoglobina, foi constatado que bebês com valores superiores a 11g/dl desenvolveram lesão de pele, ou seja, ter ou não anemia não influenciou ao aparecimento de lesão. Estudo realizado sobre o desenvolvimento de lesões por pressão mostrou que marcadores bioquímicos estão diretamente relacionados ao aparecimento de lesões, portanto, a vigilância dos níveis de hemoglobina é importante e interfere na recuperação, nutrição e oxigenação da pele^(18)^.

Embora o uso da fototerapia também não tenha mostrado relação com o aparecimento e número de lesões, é preciso destacar que este não se trata apenas de um fator de risco, mas sim, um fator que pode dificultar o tratamento quando já existe a lesão.

A hiperbilirrubinemia acomete entre 60 a 70% dos recém-nascidos à termo e entre 80 a 90% dos prematuros, tornando necessário o tratamento de fototerapia. Trata-se de uma terapia não invasiva que age diretamente na pele, quanto maior a área exposta maior é sua eficácia. Entre os cuidados, incluem-se distância de 30cm a 50cm entre a lâmpada e o bebê, conforme o tipo de lâmpada; proteção ocular; monitorização da temperatura; controle de peso; controle de umidade das mucosas e turgor da pele; mudanças de decúbito; não utilização de produtos tópicos ou emolientes. Esses cuidados são importantes para prevenir lesões de retina, queimadura, desidratação, alterações de temperatura, erupções cutâneas e eritema durante o tratamento^(19,20)^.

Um estudo apontou que o uso de emolientes na pele ressecada, com descamação ou fissuras, é importante para prevenir perda transepidérmica de água, remover adesivos e realizar higiene corporal com cremes de barreira^(5)^. Contudo, esses cuidados devem ser adiados quando o recém-nascido se encontra em fototerapia, pois favorecem o desenvolvimento de lesões.

A maioria dos recém-nascidos com lesão de pele não apresentou um escore considerado de dor na escala NIPS, entretanto, os prematuros não apresentam capacidade de modulação, inibição e redução da dor. Eles percebem a dor de forma mais intensa do que adultos e essa dura um tempo após o término de estímulos estressantes. A prevenção de lesões de pele que causam dor é crucial para o bom desenvolvimento^(21)^.

Um estudo realizado sobre práticas de avaliação de dor destacou que a utilização de escalas auxilia na detecção precoce e prevenção de alívio da dor. Diante de uma situação estressante e que causa dor, o estudo sugeriu o uso de redes para posicionamento, mobilização em decúbito lateral e ventral, favorecendo o alinhamento e a posição flexora, toque facilitado, uso de sacarose 25%, sucção não nutritiva, posição canguru, aleitamento materno, redução de luz, ruídos e estímulos^(21)^.

Com respeito ao tempo de hospitalização, foi verificado um período superior a 30 dias para os que apresentaram três lesões. Estudos mostram que as lesões de pele são responsáveis por aumentar o período de internação em unidades neonatais em cerca de 37% a 40%^(22,23)^.

Pela correlação de Pearson foram observados resultados preocupantes para o maior escore da ECPRN e o maior número de dias para cicatrização da lesão e início tardio da dieta. Sabe-se que, a imaturidade gastrointestinal dificulta a oferta e o aproveitamento de nutrientes por via enteral. No entanto, o início precoce de dieta trófica estimula a motilidade desse sistema e reduz eventos adversos. O leite materno é sempre a principal escolha, pois favorece o desenvolvimento, reduz enterocolite necrosante, sepse e outras complicações da prematuridade^(24)^.

Ademais, as lesões de pele são consideradas porta de entrada para diversos microrganismos, logo, quanto maior o tempo de exposição da pele não íntegra, maiores as chances de eventos adversos acontecerem e aumentar o tempo de hospitalização^(25)^.

A avaliação da pele do recém-nascido é considerada complexa, e quando o bebê é prematuro extremo, sua pele fina e gelatinosa, torna as lesões menos notáveis, principalmente ao se encontrar pletórico. Desse modo, os cuidados com a pele desses bebês tornam-se importantes para zelar pela proteção da sua integridade e por sua função termorreguladora, influenciando diretamente nos indicadores de qualidade da assistência, visto que, a presença de uma ou mais lesões na pele colabora para o aumento das taxas de infecção e outras complicações no período de hospitalização^(1,26)^. A estrutura subcutânea reduzida da pele de recém-nascidos prematuros e de baixo peso, muitas vezes, não é diferenciada dos demais em relação aos cuidados, colaborando para o aumento da suscetibilidade às lesões^(26)^.

Por constatar a presença de lesões de pele entre recém-nascidos hospitalizados, a equipe de enfermagem assume papel importante na prevenção, manutenção e reestruturação da pele. Nessa tríade assistencial, deve-se incluir a mudança de decúbito, manutenção da temperatura, higiene cuidadosa, uso de emolientes, cuidados com a administração de medicamentos, curativos, proteção contra o atrito dos tubos, sondas e demais equipamentos necessários para recuperação do bebê^(27)^. Cabe ao enfermeiro a elaboração de protocolos, bem como, a manutenção dessas práticas na rotina diária de cuidados em uma unidade neonatal.

### Limitações do Estudo

Como limitação da pesquisa, pode-se referir a incompletude das anotações da equipe de enfermagem no prontuário eletrônico, visto que, alterações nas condições da pele foram identificadas pela ECPRN, contudo, não estavam descritas na evolução de enfermagem. Outro fator limitador foi o preenchimento da escala de NIPS, acarretando a omissão de informações sobre a dor do recém-nascido que apresentou lesão de pele. Desse modo, recomenda-se que a equipe de enfermagem se conscientize sobre a relevância do registro das informações sobre os recém-nascidos hospitalizados de forma completa e adequada, visto a premência em garantir uma prática assistencial baseada em suas reais necessidades, contribuindo para a prevenção e tratamento das lesões de pele.

## Avanços Para Área de Enfermagem

O estudo foi importante para área da enfermagem por fornecer conhecimento sobre os fatores e características que podem aumentar as chances de um recém-nascido desenvolver lesão de pele, bem como, a necessidade de conhecer as particularidades anatômicas e fisiológicas da pele do prematuro, para que assim, os enfermeiros possam identificar os riscos precocemente e intervir de forma oportuna, visando a diminuição de sua ocorrência, qualidade assistencial, menor tempo de hospitalização, de morbimortalidade, de custos hospitalares e sofrimento para os recém-nascidos e suas famílias.

## CONCLUSÃO

O estudo mostrou frequência baixa a moderada de lesões de pele entre recém-nascidos hospitalizados, sendo que, mais da metade apresentaram mais de um tipo de lesão, predominando as dermatites e as lesões por pressão. A maioria dos bebês tinham peso ao nascer adequado e escores de Apgar >7, entretanto, a maior diversidade de lesões acometeu prematuros extremos e moderados. Recém-nascidos que não utilizaram nutrição parenteral apresentaram maiores chances de desenvolver lesões; permanência em fototerapia, presença de anemia e a utilização do maior número de antibióticos não influenciaram o aparecimento de lesões. Os bebês com três lesões permaneceram hospitalizados por mais de 30 dias.

Com base no texto fornecido, algumas implicações para o serviço de enfermagem podem ser identificadas: a equipe de enfermagem deve implementar práticas de prevenção de lesões de pele em recém-nascidos, desenvolvidas e seguidas rigorosamente, especialmente em UTI neonatal. Também é crucial realizar avaliação contínua da condição da pele dos recém-nascidos hospitalizados, permitindo intervenções precoces, bem como desenvolver protocolos e educação continuada para equipe multiprofissional com rastreamento precoce dos fatores de risco. Dada a relação entre o número de lesões e o tempo de cicatrização, a equipe de enfermagem deve estar preparada para gerenciar complicações decorrentes de múltiplas lesões, incluindo um plano de cuidados específico para cada recém-nascido com duas ou mais lesões.

Portanto, compreende-se que há necessidade de aprimorar o cuidado multiprofissional no que se refere as particularidades do bebê hospitalizado, que incluem as práticas e condutas realizadas diariamente para prevenção e manutenção da integridade da pele, visto a relevância da qualidade e da humanização da assistência, segurança do paciente, redução do tempo de hospitalização e dos custos hospitalares.

Ademais, a utilização da ECPRN mostrou-se importante, devendo ser utilizada como aliada no cuidado de enfermagem, pois auxilia na identificação precoce de lesões cutâneas, bem como na sua prevenção. Assim, sugere-se a realização de novos estudos sobre essa temática, com a finalidade de aprofundar o conhecimento e aprimorar os protocolos assistenciais em neonatologia.
